# Pancreatic Stone Protein Predicts Postoperative Infection in Cardiac Surgery Patients Irrespective of Cardiopulmonary Bypass or Surgical Technique

**DOI:** 10.1371/journal.pone.0120276

**Published:** 2015-03-20

**Authors:** Holger J. Klein, Adam Csordas, Volkmar Falk, Ksenija Slankamenac, Alain Rudiger, Felix Schönrath, Hector Rodriguez Cetina Biefer, Christoph T. Starck, Rolf Graf

**Affiliations:** 1 Division of Cardiovascular Surgery, University Hospital Zurich, Zurich, Switzerland; 2 Department of Cardiology, University Heart Center Zurich, Zurich, Switzerland; 3 Division of Visceral and Transplant Surgery, University Hospital Zurich, Zurich, Switzerland; 4 Division of Anesthesiology, Cardiosurgical Intensive Care Unit, University Hospital Zurich, Zurich, Switzerland; Sapienza University of Rome, ITALY

## Abstract

**Introduction:**

We investigated the role of pancreatic stone protein (PSP) in predicting the occurrence of infection in the postoperative course of cardiac surgery patients. Several biomarkers indicating the presence of inflammation and infection are available in the clinical routine; yet, their utility in the postoperative course of patients following cardiac surgery remains uncertain. Moreover, cardiopulmonary bypass, also referred to as “on-pump surgery”, increases the susceptibility to an exaggerated inflammatory state. However, the impact of such extracorporeal circulation on circulating PSP levels remains poorly understood.

**Methods:**

In a prospective cohort of unselected patients undergoing cardiac surgery, we set out to elucidate the diagnostic accuracy of serum PSP levels as opposed to canonical biomarkers (CRP, WBC) of inflammation to discriminate between the presence of infection and surgical trauma,. In addition, we investigated whether the biomarkers were influenced by the surgical technique employed, i.e. on-pump vs. off-pump and minimally invasive surgery vs. sternotomy. Levels of circulating PSP and routine inflammatory biomarkers (CRP, WBC) were measured in samples taken from 120 patients at baseline as well as at postoperative day 1–3.

**Results:**

Univariate analysis showed that among the biomarkers investigated, only PSP levels had discriminatory power to differentiate infection from surgical trauma in the postoperative course of the entire cohort of patients following cardiac surgery. With regard to cardiac surgical interventions, there was no significant association between the absence or presence of extracorporeal circulation and PSP levels. However, there was a significant difference in the slope of the rise of postoperative PSP between minimally invasive surgery as opposed to patients subjected to sternotomy.

**Conclusion:**

In an unselected population of cardiac surgery patients, post-operative serum PSP levels were significantly associated with the presence of infection in both the on-pump and off-pump setting. Of note, the surgical technique employed (sternotomy vs. minimally invasive approach) had a significant impact on postoperative PSP levels.

## Introduction

Despite decades of intensive research and constant evolution of clinical experience, mortality and morbidity associated with sepsis remains substantial and is higher than that associated with heart failure or many cancers in the range of up to 18–30% [1, 2]. A major factor in the poor clinical outcome of patients presenting with sepsis even under the best possible care in the ICU is the lack of reliable diagnostic tools in the timely identification of patients needing emergency medical care [3]. In a clinical syndrome similar to sepsis, albeit in the absence of bacterial infection as an underlying trigger, designated as the systemic inflammatory response syndrome (SIRS), release of endogenous danger signals or so-called damage-associated molecular patterns (DAMPS) as a result of tissue injury activates a set of pro-inflammatory circuits reminiscent of those described in septic shock [4, 5]. SIRS might present in a subclinical form or progress to a fulminant state with multiorgan failure. In patients undergoing cardiac surgery, use of extracorporeal circulation is inevitably associated with the occurrence of some degree of exaggerated inflammation that is added to the surgical stress inherent to the procedure itself [6, 7]. The degree of such sterile inflammation is associated with an increased rate of morbidity and hence poor clinical outcome [8, 9]. Consequently, there is an urgent need for adequate biomarkers to differentiate patients with postoperative infection from those with reactive inflammation at an early time point. This may help clinical decision making at the incipient stage of the disease, thus potentially increasing the opportunity for effective therapeutic intervention. The major challenge clinicians are facing in the development of accurate diagnostic and prognostic biomarkers in septic patients compared to other clinical emergencies relates to the complexity of the underlying disease process. Consequently, more than 3000 types of biomarkers have been suggested as potential diagnostic tools in septic patients, while, by comparison, there are only about 15 biomarkers available for the diagnosis of acute myocardial infarction [3].

However, a clear diagnostic algorithm for timely identification of patients at risk for a life threatening maladaptive inflammatory response has not yet emerged. For instance, implementation of procalcitonin (PCT)-based protocols has proven useful in supporting clinical decision making with regard to tailoring the duration of antibiotic treatment in septic ICU patients but does not allow for early diagnosis or prognostic stratification of these patients [10]. Circulating levels of pro-inflammatory cytokines help identify patients at risk of early deterioration, but their usefulness in clinical diagnosis is limited by their narrow time frame of expression [11]. Against this background, the emergence of pancreatic stone protein/regenerating protein (PSP/reg) as a novel diagnostic and prognostic biomarker in a broad range of septic ICU patients is a promising development [12–14]. Originally described as a protein constitutively secreted by pancreatic acinar cells, insights from recent studies on the role of PSP as a biomarker for SIRS and sepsis in a heterogeneous patient population has led to an appreciation of a much broader functional scope of this protein. The initial circulating levels of this protein enable prognostic assessment of the evolution of the disease in patients suffering from SIRS or sepsis. For instance, in a prospective study it was shown that PSP, measured within 24 h after admission to ICU, performed much better in predicting the risk for mortality in patients presenting with severe sepsis or septic shock compared to canonical markers of ongoing inflammation [14]. Similarly, a role for predicting outcome in patients with ventilator-associated pneumonia or COPD with superimposed bacterial infection has been suggested [13]. In another study, measures of PSP were closely correlated with the severity of infection in trauma patients [15]. Moreover, data suggest that initial circulating PSP levels might be clinically useful in distinguishing between patients suffering from sepsis and those with a non-infective inflammatory response [14].

The molecular mechanism by which PSP reacts to infection is not fully understood. In contrast to many products of the pancreatic acinar cell, which are stimulated by hormones, the synthesis and secretion of PSP, and of a few other proteins, is also stimulated by cytokines and other signals. Hence, the pancreas is also recognized as an acute phase organ [16]. These proteins are found in pancreatic juice but are also diverted to the interstitium and end up in the bloodstream. We have previously shown by FACS-analysis of blood-derived leukocytes, that PSP interacts with granulocytes and propagates their activation (15). A detailed mechanism of how PSP affects the innate immune system remains to be shown.

In the present study, we sought to determine whether at an incipient stage of the disease, PSP predicts the onset of infection in the early postoperative course of patients undergoing cardiac surgery. Furthermore, we wanted to elucidate the impact of extracorporeal circulation and of surgical techniques on circulating PSP levels and the ability of PSP to discriminate sterile systemic inflammation from infection. To pursue these aims, we prospectively measured baseline and early postoperative serum PSP levels at day 1–3 in 120 unselected patients who underwent cardiac surgery and closely monitored their postoperative course with respect to onset of clinically manifest infection.

## Materials and Methods

### Participants

Approval for the current study was obtained from the Ethics Committee of the University of Zurich. Calculated sample size was estimated a priori by GPOWER 3.1 [17] resulting in a requested number of approximately 110 patients (given α = 0.05, power = 0.9, effect size = 0.25). Between May and December 2012, after giving written informed consent, each patient older than 18 years of age (N = 145) awaiting elective cardiac surgery was included, in the study on the day of admission to hospital (usually one day before surgery). Exclusion criteria were age < 18 years and preexistent infections, such as urinary tract infections, endocarditis, and pneumonia or wound infections. Out of the 145 patients, 120 patients who fulfilled the inclusion criteria were enrolled in this prospective, single-center cohort study.

### Measurement of serum/plasma biomarker concentration

Biomarkers of concern for the current study (PSP, CRP, WBC) were obtained from the pre- and postoperative routine blood samples. CRP and WBC were determined by standard procedures. PSP was measured as described previously [15]. PSP measurements were performed by an experienced technician at the University Hospital of Zurich. All clinicians involved in the study were blinded to PSP results whereas they were aware of WBC and CRP values.

### Preoperative data

At admission, baseline blood samples were taken for analysis of PSP, CRP, WBC and other preoperative routine parameters. Data on age (years), gender (male/female), height (in cm), weight (in kg), BMI, diabetes mellitus (y/n) and the Euroscore II (%) were collected.

### Operative data

The different procedures were classified under five categories (isolated bypass surgery, isolated valve surgery, aortic surgery, combined procedures, others). The surgical approach was differentiated in sternotomy versus minimally invasive procedures. Further variables were heart-lung machine (HLM) (y/n), time of extracorporeal circulation (EEC in minutes), time of cardiac ischemia (in minutes) and total surgery time. Intraoperative measurement of PSP was not performed.

### Postoperative data

First postoperative blood for quantifying the postoperative levels of PSP, CRP and WBC was obtained within 24 hours after each procedure followed by further daily blood samples until patient’s discharge. Whenever postoperative infections or sepsis were suspected, we used the definition of infection and sepsis as defined in Levy et al. [18].

### Endpoints

- Primary endpoint: time course of PSP serum levels before and after elective cardiac surgery (baseline, 24h/48h/72h postoperative).
- Secondary endpoints:
  - time course of PSP serum levels in absence/presence of infection compared with time course of routine markers of inflammation (CRP, WBC)
  - time course of PSP serum levels in absence/presence of cardiopulmonary bypass compared with time course of routine markers of inflammation (CRP, WBC)
  - time course of PSP levels as related to the surgical technique employed (sternotomy/minimally invasive approach) compared with time course of routine markers of inflammation (CRP, WBC)

### Statistical analysis

Discrete values are expressed as counts (percentages) and continuous variables as means (SD) or medians (IQR) as appropriate. Loge transformation of PSP, CRP and WBC values was carried out to reach normal distribution of data for parametric analysis. Comparison of baseline levels of inflammatory markers investigated (PSP, CRP, WBC) with clinical characteristics was carried out with one-way ANOVA. Receiver operating characteristics analysis was employed at postoperative day 1–3 for evaluation of the diagnostic performance of PSP, CRP and WBC for prediction of the clinical outcome infection. Values of the area under the curve (AUC) are reported with corresponding 95% CI. Cut-off points for PSP levels at postoperative day 2 were selected giving equal weight to sensitivity and specificity. To delineate the time course of PSP following cardiac surgery, a linear mixed effects regression model with random intercepts was employed with determination of an interaction between group (infection yes/no) and time. In a second step, a split-plot ANOVA design was employed to adjust for associations of PSP levels with baseline characteristics (age, diabetes). Univariate binary logistic regression analysis was used to test PSP, CRP and WBC as predictors for the clinical outcome postoperative infection. All tests were two tailed; p < .05 was considered significant. Data were analyzed using statistical software (Statistical Package for Social Sciences, Version 20 for Macintosh; SPSS; Chicago, Illinois).

## Results

### Baseline characteristics and outcome of the study population

Baseline characteristics of the whole study population are given in Table I. The median age of the patients was 66.5 years. Of these, 27% were female. The median length of hospital stay was 8.5 days. A total of 20 patients (16.7%) had known diabetes mellitus II. Overall, 40 patients (33.3%) received isolated coronary artery bypass grafting (CABG) while 41 patients (34.2%) received isolated valve surgery (including replacement and reconstruction of the aortic or mitral valve). In the former group, 22.5% of surgery was undertaken without the use of cardiopulmonary bypass (off-pump). In 26 patients (21.7%), combined heart surgery including CABG and valve surgery was performed. Among the entire study population, in 17 patients (14.2%) a minimally invasive surgical technique was employed. The median stay in the ICU was 23 hours. In the ICU, 95% of patients were in need of vasopressors signifying the presence of some degree of SIRS in the vast majority of patients. The early postoperative course was uneventful in 102 (85%) patients while a diagnosis of infection was established in 18 patients (15%). Of these, 3 patients fulfilled the current diagnostic criteria of sepsis. In the great majority of patients, the focus of infection was either in the urinary or respiratory tract with a minority of patients suffering from peripheral wound infection. There were two documented cases of sternal wound infections and one patient was diagnosed with mediastinitis. One patient died in the ICU secondary to postoperative heart failure resulting in an overall mortality rate of 0.8%.

**Table I:** Patient- and surgery-related characteristics (n = 120).

Age in years (median, IQR)	66.5 (54.2–75.0)
Gender male/female (n, %)	88/32 (73%/27%)
BMI in kg/m^2^ (median, IQR)	26.3 (23.6–30.4)
Diabetes mellitus yes/no (n, %)	20/100 (16.7%/83.3%)
EuroScore II (median, IQR)	3.1% (1.5%- 5.3%)
HLM yes/no (n, %)	93/27 (77.5%/22.5%)
Time of ECC in min (mean, SD)	143.7 ± 56.6
Time of ischemia in min (mean, SD)	97 ± 45
Total time of surgery in min (mean, SD)	245.6 ± 105
Total stay in ICU in hours (median, IQR)	23 (19–40.5)
Total stay in hospital in days (median, IQR)	8.5 (7–12)
Type of operation (n, %)	
Isolated CABG	40 (33.3%)
Isolated valve replacement/reconstruction	41 (34.2%)
Isolated aortic surgery	1 (0.8%)
Combined surgery	26 (21.7%)
Others	12 (10.0%)
Surgical approach (n, %)	
Sternotomy	97 (80.8%)
Mini thoracotomy	17 (14.2%)
Others	6 (5.0%)
Infection yes/no (n, %)	18/102 (15%/85%)
Pneumonia (n, %)	8 (44.4%)
Urinary tract infection (n, %)	3 (16.6%)
Peripheral wound infection (n, %)	4 (22.2%)
Sternal wound infection (n, %)	2 (11.1%)
Mediastinitis (n, %)	1 (5.6%)
Sepsis yes/no (n, %)	3/117 (2.5%/97.5%)
Mortality yes/no (n, %)	1/119 (0.8%/99.2%)

*SD* = *standard deviation*

*IQR* = *interquartile range*

### Association between inflammatory markers and baseline characteristics

**Fig 1:**
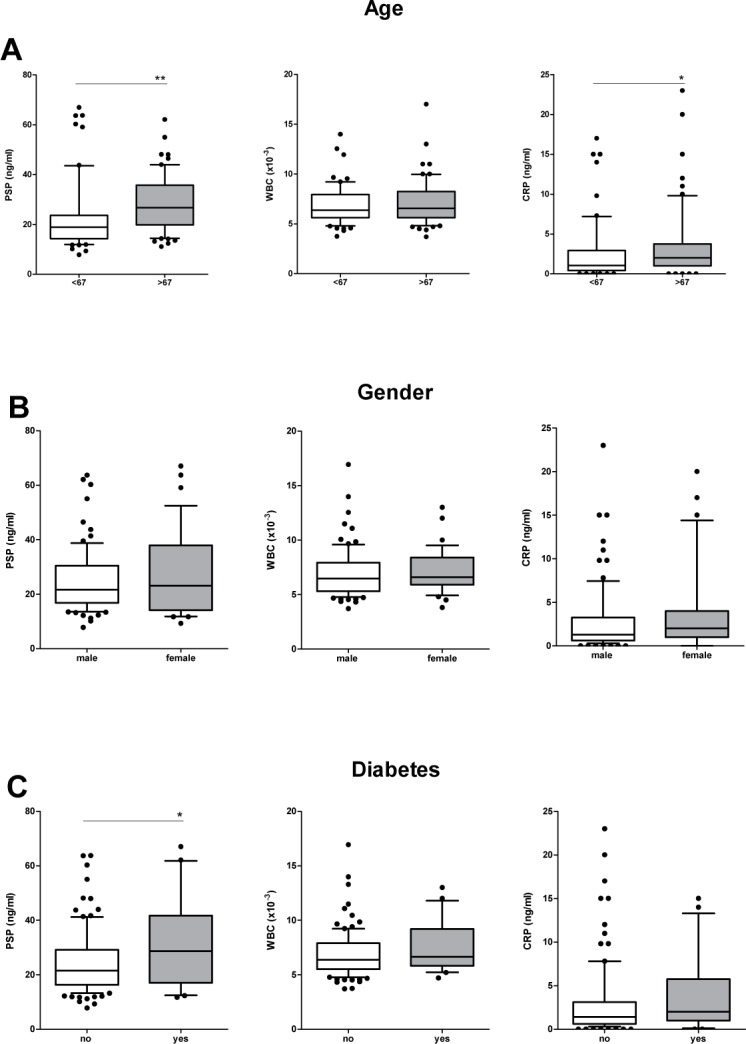
Association of PSP, CRP and WBC levels with age (A), gender (B) and diabetes (C). Shown are values at baseline (preoperative values). The groups were compared by one-way ANOVA. Asterisks indicate significant differences (*p<0.05; **p<0.01).

Serum levels of biomarkers are shown in Fig. 1. Of note, at baseline PSP was significantly higher in patients with diabetes mellitus II as opposed to non-diabetic study participants (p = 0.008). There was no correlation between PSP levels and adiposity (BMI >30) (p = 0.324). However, PSP levels were significantly higher in elderly patients above the median age of 67 years (p = 0.01). The difference of PSP among diabetic patients lost statistical significance after adjusting for age. There was no significant difference in the distribution of WBC and CRP levels in elderly or diabetic patients.

### Time course of inflammatory markers in patients after cardiac surgery as related to postoperative infection and sepsis

**Fig 2:**
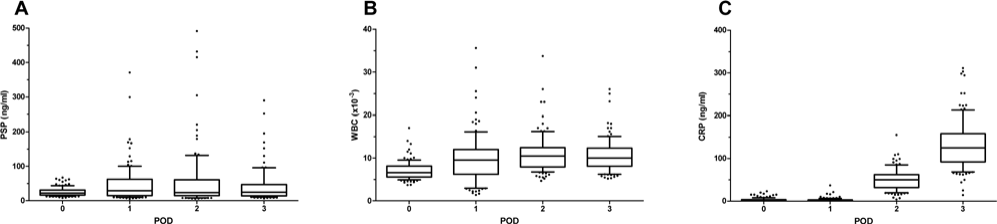
Time course of biomarkers (A-C) following cardiac surgery. All inflammatory markers investigated showed a statistically significant rise in the course of postoperative day 1–3 (p<0.05, determined by a linear mixed effects regression model).

**Fig 3:**
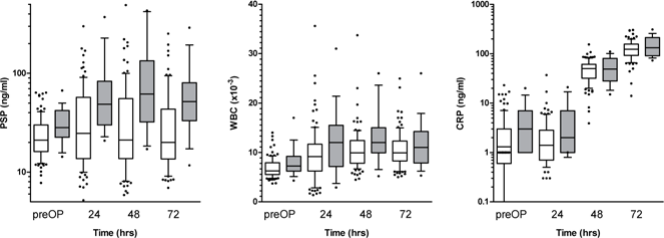
Discriminatory power of biomarkers for postoperative infection. PSP levels showed a significantly stronger postoperative rise at day 1–3 in patients receiving a diagnosis of infection during their hospital stay as opposed to patients exhibiting an uneventful course. (p<0.05 for interaction, determined by a linear mixed effects regression model). Other biomarkers (CRP and WBC) failed to differentiate infection from postoperative inflammatory power.

Univariate analysis using a linear mixed effects regression model showed a statistically significant increase of PSP levels within 72 h following cardiac surgery (p < 0.05) in the entire study population (Fig. 2). Similarly, canonical pro-inflammatory markers such as WBC and CRP increased markedly with 72 h after cardiac surgery (p<0.001 for CRP, p<0.001 for WBC) (Fig. 2); in the postoperative distribution of PSP levels, there was a significant interaction between developing an infection and time, signifying a significantly steeper increase in PSP levels in patients developing infection as opposed to those exhibiting an uneventful course (p value for interaction = 0.02) (Fig. 3). However, neither of the other parameters had the ability to differentiate infection from surgical inflammation (p-value for interaction = 0.4 for WBC and 0.29 for CRP) (Fig. 3). In order to further substantiate the discriminatory power of PSP to detect the presence of infection, we employed a multivariate split plot ANOVA design adjusting for age and diabetes. While CRP and WBC again lacked any discriminatory power in identifying the presence of infection, PSP levels proved to be significantly higher in patients with infection in the adjusted analysis (p = 0.015). In the cohort of patients with postoperative infection, there were three cases fulfilling the criteria of sepsis as outlined above. Again, employing univariate analysis, PSP levels were significantly higher in septic patients during 72 hours following cardiac surgery as opposed to patients exhibiting an uneventful postoperative course (p = 0.001) while CRP and WBC levels were increased irrespective of the absence or presence of infection. To underline the role of PSP in differentiating infection PSP levels in patients requiring postoperative antibiotics are shown in comparison to those with an uneventful course (S1 Fig.).

### Association of biomarkers with type of surgery

**Fig 4:**
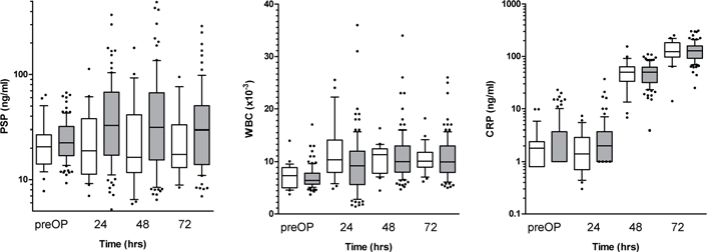
Time course of biomarkers as related to the absence or presence of cardiopulmonary bypass. All inflammatory markers investigated showed a steeper rise in patients subjected to cardiopulmonary bypass without reaching statistical significance. (p>0.05 for interaction for all biomarkers, determined by a linear mixed effects regression model).

**Fig 5:**
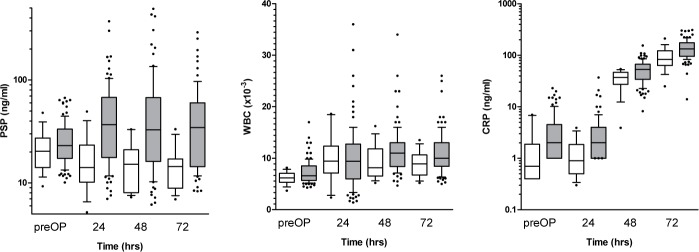
Time course of biomarkers as related to the surgical technique employed. PSP levels showed a significantly steeper rise in the postoperative course of patients subjected to sternotomy compared to those receiving minimally invasive surgery (p<0.05 for interaction, determined by a linear mixed effects regression model). There was no significant difference in the postoperative levels of CRP and WBC between the two cohorts of patients.

Focusing on patients undergoing off-pump surgery in comparison to those exposed to cardiopulmonary bypass (on-pump surgery), there was no significant difference in PSP, CRP or WBC levels at postoperative day 1–3 (p>0.05 for all biomarkers) (Fig. 4). To look more closely on the impact of surgical trauma as related to postoperative dynamics of the biomarkers, we stratified patients according to type of surgery (minimally invasive approach versus sternotomy). Patients undergoing sternotomy had a significantly steeper rise of postoperative PSP (p≤0.001) as opposed to those subjected to a minimally invasive approach. The type of surgical technique had no significant impact on the postoperative distribution on CRP and WBC levels (p>0.05) (Fig. 5).

### Association of PSP levels with clinical outcomes

**Fig 6:**
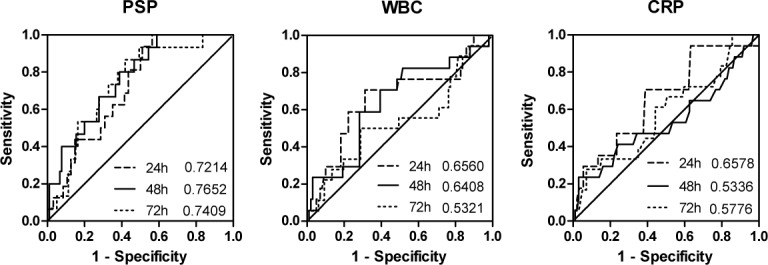
Receiver operating characteristics curve to predict infection for PSP (A), CRP (B) and WBC (C) at postoperative 1–3. A cutoff of 48.1 ng/ml for PSP levels at postoperative day 2 reveals a sensitivity of 64% and a specificity of 70%. PLR (positive likelihood ratio) = 2.1. NLR (negative likelihood ratio) = 0.51.

To further corroborate the potential predictive ability of PSP for the presence of infection in cardiac surgery patients, we employed ROC curve analysis for PSP at postoperative day 1–3. Fig. 6 illustrates the ROC curves and indicates the area under curve for PSP, WBC and CRP. The highest sensitivity and specificity was achieved by PSP at postoperative day 2 (AUC = 0.765, CI = 0.621–0.877). Traditional inflammatory markers showed basically no predictive ability for infection (AUC for CRP at postoperative day 2 = 0.534, AUC for WBC at postoperative day 2 = 0.641). Giving equal weight to both sensitivity and specificity, we selected 48.1ng/ml for PSP. With this cutoff point we were able to predict yet clinically unapparent postoperative infection at postoperative day 2. Moreover, in univariate logistic regression analysis PSP at day 2 significantly predicted the clinical outcome postoperative infection (OR = 2.5, p = 0.001), while canonical biomarkers for infection (WBC, CRP) lacked any discriminatory ability in this regard.

## Discussion

In this pilot study, our aim was to investigate the diagnostic accuracy of PSP for the presence of postoperative infections in an unselected cohort of patients undergoing cardiac surgery. Although substantial evidence points towards a potential value of PSP as a novel sepsis biomarker with the ability to identify and risk-stratify patients in a broad range of clinical spectra, its role in surgical patients remains unclear. Of note, cardiac surgery entailing the use of cardiopulmonary bypass was repeatedly associated with some degree of systemic inflammation, which is reflected by an increase in circulating levels of pro-inflammatory biomarkers and clinical picture resembling septicemia [19]. Consequently, it is particularly challenging to establish a diagnosis of infection beyond reactive inflammation secondary to surgical stress in the postoperative setting. In the present study, we found a significant rise in the levels of both PSP and canonical inflammatory markers (CRP, WBC) following cardiac surgery. However, there was a significant rise of PSP levels in patients diagnosed with infection during their postoperative course over the following 72 h compared to those whose postoperative course was uneventful. Importantly, the same aforementioned time course of inflammatory markers was observed irrespective of the use of cardiopulmonary bypass. Concerning the surgical technique (sternotomy versus minimally invasive approach), we found a significant difference in the slope of postoperative PSP values with a markedly steeper increase in those exposed to sternotomy. However, PSP retained its ability to detect infection in the entire cohort studied. Overall, these data suggest that although surgical trauma has a significant impact on PSP levels, the slope of the rise in PSP levels more specifically mirrors infectious conditions above a reactive increase of this secretory protein upon surgical trauma. Canonical inflammatory markers such as circulating levels of CRP and WBC, on the other hand, appear to primarily reflect surgical trauma, without enabling identification of the presence of infection and cannot be used for determining the presence or absence of infection. It should be emphasized that the exact origin and essential triggers for PSP release are far from clear. Previous studies suggest that PSP plays a role in activating neutrophils and aggregating bacteria. Of note, PSP has been demonstrated to bear a high degree of structural homology with lectins suggesting an as yet under-appreciated role for PSP during bacterial infection [20]. Consistent with this finding, the obervation of a much steeper rise of PSP in patients suffering postoperative infection provides further evidence for a role of PSP as a predictor of infection at incipient stages of the disease. Although initially identified in pancreas-derived calcified concrements [21], in the last few years it became increasingly evident that PSP is expressed in a much broader variety of tissues including the stomach and small intestine [22]. Previous studies demonstrated that levels of expression of PSP are drastically increased in response to various types of inflammatory conditions in the absence of acute pancreatitis. Compared to traditional biomarkers of inflammation (such as WBC and CRP), PSP emerged not only as a promising biomarker for sepsis but also as a predictor for clinical outcomes including sepsis or death at the ICU [23]. This finding ties in well with our finding of PSP primarily reflecting infection rather than non-infectious states of systemic inflammation. Previous studies mainly focused on the role of PSP with regard to infection and its predictive power for clinical outcomes in septic patients. However, it remained unclear whether the increased PSP levels were due to activation of infection pathways or rather serve as a surrogate parameter for tissue hypoperfusion [24]. Given that cardiopulmonary bypass is inevitably associated with some degree of unfavorable tissue perfusion our finding of PSP levels showing a similar time course in both the off-pump and on-pump setting clearly suggests that non-infectious systemic inflammation is associated with only a moderate rise in PSP levels [25]. The presence of clinically overt infection, however, was associated with a marked increase in PSP irrespective of preceding surgical trauma. Given that 75% of study participants were in need of postoperative vasopressors, it can be assumed that a large majority of patients indeed suffered from some degree of postoperative inflammatory trauma. Consequently, in our study PSP retained discriminatory power for infection even against the background of sterile systemic inflammation. This finding could be confirmed by various approaches of statistical analysis (linear mixed effects model, split-plot ANOVA). In this study, we employed 3 consecutive measurements of postoperative PSP levels at 24 h, 48 h and 72 h. A closer look at the time course of PSP shows that the curves deviate already after 24 h with a peak of PSP after 48 h in patients with postoperative infection. After 72 h, there was no further increase of PSP levels. Altogether, it would appear that a time frame between 24 h and 48 h following cardiac surgery would be ideal for early diagnosis of infection, as yet clinically unapparent, in cardiac surgery patients and associated reactive inflammation.

ROC curve analysis showed the greatest sensitivity and specificity at 48 h in the postoperative course, with a cut-off point of 48.1 ng/L for the clinical outcome infection. Although PSP performed best among the inflammatory markers investigated given the rather moderate AUC, the overall discriminatory power in terms of increasing the post-test probability for infection remains suboptimal. Another important observation relates to the association of age and diabetes with baseline PSP values. Elderly patients (above the median age of 67 years) were found to have significantly higher PSP levels at baseline than their younger counterparts. A similar observation could be made in patients with suspected or established diabetes mellitus [26]. The relevance of age as well as the impact of age-related comorbidities on PSP levels need to be investigated in future clinical trials. The finding of an association of PSP levels with diabetic status suggests a pro-inflammatory state in this cohort of patients, which is in agreement with previous studies. However, PSP proved of value for predicting infection in the entire cohort studied independent of a diagnosis of diabetes at baseline. There are further limitations of the study. First of all, our pilot study on the time course of PSP in cardiac surgery patients and its relationship with clinically apparent infection needs to be considered as hypothesis generating and awaits confirmation in future studies. Moreover, given the single-center design of our study, there is no external validation of our data. In this study, it was our aim to enroll an unselected cohort of cardiac surgery patients to capture the real-life setting as closely as possible. However, given the small sample size, it was not feasible to control for multiple baseline and surgery- related characteristics, and to elucidate the time course of postoperative PSP levels as related to the type of infection. Consequently, the impact of different comorbidities on PSP levels at baseline as well as in the postoperative course remains unclear. In future trials, a major focus should be put on the impact of potential clinical confounders on the distribution of PSP levels. Nevertheless, our study corroborates the potential of PSP to differentiate bacterial infection from reactive inflammation in a cohort of patients subjected to a substantial degree of inflammatory stimulation and hence underpins the clinical relevance of this novel biomarker for detection of infection in cardiac surgery patients. Given the high morbidity associated with a postoperative course of cardiac surgery complicated by infection, biomarkers that enable early clinical decision making in this cohort of patients are clearly warranted.

Overall, we conclude that levels of PSP at postoperative day 1–3 possess discriminatory properties for infection in the context of an unselected cohort of patients undergoing cardiac surgery. This finding is evident irrespective of the surgical technique or use of cardiopulmonary bypass. Consequently, PSP is poised well as a promising biomarker at the crossroads of reactive inflammation and infection. However, at present, thorough clinical assessment of patients by an experienced and preferably multidisciplinary team remains the mainstay in the evaluation of patients deemed at risk for sepsis. Future clinical studies are required to further assess whether PSP has a future role in clinical decision making in the postoperative course of cardiac surgery patients.

## Supporting Information

S1 Fig Time course of circulating PSP levels in patients requiring postoperative antibiotics over the course of 12 days (p < 0.05 after 24 hours postoperative). (EPS)

## References

[1] Martin GS, Mannino DM, Eaton S, Moss M. The epidemiology of sepsis in the United States from 1979 through 2000. N Engl J Med. 2003;348(16):1546–54. 12700374 10.1056/NEJMoa022139

[2] Angus DC, van der Poll T. Severe sepsis and septic shock. N Engl J Med. 2013;369(21):2063 10.1056/NEJMc1312359#SA3 24256390

[3] Pierrakos C, Vincent JL. Sepsis biomarkers: a review. Critical care. 2010;14(1):R15 10.1186/cc8872 20144219 PMC2875530

[4] Zhang Q, Raoof M, Chen Y, Sumi Y, Sursal T, Junger W, et al Circulating mitochondrial DAMPs cause inflammatory responses to injury. Nature. 2010;464(7285):104–7. 10.1038/nature08780 20203610 PMC2843437

[5] Chen GY, Nunez G. Sterile inflammation: sensing and reacting to damage. Nature reviews Immunology. 2010;10(12):826–37. 10.1038/nri2873 21088683 PMC3114424

[6] Holmes JHt, Connolly NC, Paull DL, Hill ME, Guyton SW, Ziegler SF, et al Magnitude of the inflammatory response to cardiopulmonary bypass and its relation to adverse clinical outcomes. Inflammation research: official journal of the European Histamine Research Society [et al]. 2002;51(12):579–86. 10.1007/pl00012432 12558191

[7] Litmathe J, Boeken U, Bohlen G, Gursoy D, Sucker C, Feindt P. Systemic inflammatory response syndrome after extracorporeal circulation: a predictive algorithm for the patient at risk. Hellenic journal of cardiology: HJC = Hellenike kardiologike epitheorese. 2011;52(6):493–500. 22143012

[8] Sablotzki A, Friedrich I, Muhling J, Dehne MG, Spillner J, Silber RE, et al The systemic inflammatory response syndrome following cardiac surgery: different expression of proinflammatory cytokines and procalcitonin in patients with and without multiorgan dysfunctions. Perfusion. 2002;17(2):103–9. 11958300 10.1177/026765910201700206

[9] Sinning JM, Scheer AC, Adenauer V, Ghanem A, Hammerstingl C, Schueler R, et al Systemic inflammatory response syndrome predicts increased mortality in patients after transcatheter aortic valve implantation. Eur Heart J. 2012;33(12):1459–68. 10.1093/eurheartj/ehs002 22285582

[10] Kopterides P, Siempos II, Tsangaris I, Tsantes A, Armaganidis A. Procalcitonin-guided algorithms of antibiotic therapy in the intensive care unit: a systematic review and meta-analysis of randomized controlled trials. Crit Care Med. 2010;38(11):2229–41. 10.1097/CCM.0b013e3181f17bf9 20729729

[11] Hotchkiss RS, Karl IE. The pathophysiology and treatment of sepsis. N Engl J Med. 2003;348(2):138–50. 12519925 10.1056/NEJMra021333

[12] Que YA, Delodder F, Guessous I, Graf R, Bain M, Calandra T, et al Pancreatic stone protein as an early biomarker predicting mortality in a prospective cohort of patients with sepsis requiring ICU management. Critical care. 2012;16(4):R114 10.1186/cc11406 22748193 PMC3580689

[13] Boeck L, Graf R, Eggimann P, Pargger H, Raptis DA, Smyrnios N, et al Pancreatic stone protein: a marker of organ failure and outcome in ventilator-associated pneumonia. Chest. 2011;140(4):925–32. 10.1378/chest.11-0018 21835904

[14] Llewelyn MJ, Berger M, Gregory M, Ramaiah R, Taylor AL, Curdt I, et al Sepsis biomarkers in unselected patients on admission to intensive or high-dependency care. Critical care. 2013;17(2):R60 10.1186/cc12588 23531337 PMC3672658

[15] Keel M, Harter L, Reding T, Sun LK, Hersberger M, Seifert B, et al Pancreatic stone protein is highly increased during posttraumatic sepsis and activates neutrophil granulocytes. Crit Care Med. 2009;37(5):1642–8. 10.1097/CCM.0b013e31819da7d6 19325491

[16] Keim V, Iovanna JL, Dagorn JC. The acute phase reaction of the exocrine pancreas. Gene expression and synthesis of pancreatitis-associated proteins. Digestion. 1994;55(2):65–72. 8187976 10.1159/000201127

[17] Erdfelder E, Buchner A, Faul F, Brandt M. GPOWER: Teststärkenanalyse leicht gemacht In: Erdfelder E, Funke J, editors. Allgemeine und deduktivistische Methodologie. Göttingen: Vandenhoeck & Ruprecht; 2004 p. 148–66.

[18] Levy MM, Fink MP, Marshall JC, Abraham E, Angus D, Cook D, et al 2001 SCCM/ESICM/ACCP/ATS/SIS International Sepsis Definitions Conference. Intensive care medicine. 2003;29(4):530–8. 12664219 10.1007/s00134-003-1662-x

[19] Cremer J, Martin M, Redl H, Bahrami S, Abraham C, Graeter T, et al Systemic inflammatory response syndrome after cardiac operations. Ann Thorac Surg. 1996;61(6):1714–20. 8651772 10.1016/0003-4975(96)00055-0

[20] Iovanna J, Frigerio JM, Dusetti N, Ramare F, Raibaud P, Dagorn JC. Lithostathine, an inhibitor of CaCO3 crystal growth in pancreatic juice, induces bacterial aggregation. Pancreas. 1993;8(5):597–601. 8302796 10.1097/00006676-199309000-00011

[21] De Caro A, Lohse J, Sarles H. Characterization of a protein isolated from pancreatic calculi of men suffering from chronic calcifying pancreatitis. Biochem Biophys Res Commun. 1979;87(4):1176–82. 111670 10.1016/s0006-291x(79)80031-5

[22] Carrere J, Figarella-Branger D, Senegas-Balas F, Figarella C, Guy-Crotte O. Immunohistochemical study of secretory proteins in the developing human exocrine pancreas. Differentiation; research in biological diversity. 1992;51(1):55–60. 1451962 10.1111/j.1432-0436.1992.tb00680.x

[23] Gukasjan R, Raptis DA, Schulz HU, Halangk W, Graf R. Pancreatic stone protein predicts outcome in patients with peritonitis in the ICU. Crit Care Med. 2013;41(4):1027–36. 10.1097/CCM.0b013e3182771193 23399938

[24] Busani S, Girardis M. PSP/reg: a new stone in sepsis biomarkers? Critical care. 2012;16(4):143 10.1186/cc11433 22856672 PMC3580713

[25] Prondzinsky R, Knupfer A, Loppnow H, Redling F, Lehmann DW, Stabenow I, et al Surgical trauma affects the proinflammatory status after cardiac surgery to a higher degree than cardiopulmonary bypass. J Thorac Cardiovasc Surg. 2005;129(4):760–6. 15821641 10.1016/j.jtcvs.2004.07.052

[26] Yang J, Li L, Raptis D, Li X, Li F, Chen B, et al Pancreatic stone protein/regenerating protein (PSP/reg): a novel secreted protein up-regulated in type 2 diabetes mellitus. Endocrine. 2014. 10.1007/s12020-014-0427-3 25234740

